# The consequences of living with a severe malocclusion: A review of the literature

**DOI:** 10.1177/14653125211042891

**Published:** 2021-09-06

**Authors:** Richard Leck, Ninu Paul, Sarah Rolland, David Birnie

**Affiliations:** School of Dental Sciences, Newcastle University, Newcastle upon Tyne, UK

**Keywords:** orthognathic surgery, orthognathic treatment, malocclusion, aetiology of malocclusion and growth, occlusal development, restorative-orthodontic interface, interdisciplinary treatment, sleep apnoea, health services and quality of life aspects, quality of life and orthodontics, risk/benefit assessment, craniofacial growth, genetics of malocclusion

## Abstract

**Aim:**

To facilitate the orthognathic shared decision-making process by identifying and applying existing research evidence to establish the potential consequences of living with a severe malocclusion.

**Methods:**

A comprehensive narrative literature review was conducted to explore the potential complications of severe malocclusion. A systematic electronic literature search of four databases combined with supplementary hand searching identified 1024 articles of interest. A total of 799 articles were included in the narrative literature review, which was divided into 10 themes: Oral Health Related Quality Of Life; Temporomandibular Joint Dysfunction; Masticatory Limitation; Sleep Apnoea; Traumatic Dental Injury; Tooth Surface Loss; Change Over Time; Periodontal Injury; Restorative Difficulty; and Functional Shift and Dual Bite. A deductive approach was used to draw conclusions from the evidence available within each theme.

**Results:**

The narrative literature review established 27 conclusions, indicating that those living with a severe malocclusion may be predisposed to a range of potential consequences. With the exception of Oral Health Related Quality Of Life, which is poorer in adults with severe malocclusion than those with normal occlusions, and the risk of Traumatic Dental Injury, which increases when the overjet is >5 mm in the permanent and 3 mm in the primary dentition, the evidence supporting the remaining conclusions was found to be of low to moderate quality and at high risk of bias.

**Conclusion:**

This article summarises the findings of a comprehensive narrative literature review in which all of the relevant research evidence within a substantive investigative area is established and evaluated. Notwithstanding limitations regarding the quality of the available evidence; when combined with clinical expertise and an awareness of individual patient preferences, the conclusions presented may facilitate the orthognathic shared decision-making process and furthermore, may guide the development of the high-quality longitudinal research required to validate them.

## Introduction

Approximately 250,000 people in the UK have a severe enough malocclusion to justify orthognathic surgery (Kumar et al., 2008). When resources are limited, it is important that we consider the financial, social, emotional and functional consequences for those who do not receive an intervention, while recognising the benefits for those that do. Since 2006, access to NHS orthodontic treatment in England and Wales has been primarily based on the Index of Orthodontic Treatment Need (Brook and Shaw, 1989). While the fundamental question remains whether the patient will benefit from treatment, professionally assessed existence of disease does not always align with the concept of illness and health from the patient’s perspective (Inglehart and Bagramian, 2002). Similarly, patients who, in the clinician’s opinion, have high levels of orthodontic need, may be submitted for orthognathic treatment despite possessing little desire to undergo invasive elective surgery.

The law requires healthcare professionals to ensure that the patient is aware of all material risks (Campbell, 2015). In addition, the concept of shared decision-making; which demands adequate knowledge, motivation and engagement from all decision-makers (Da Silva, 2012), refers to the importance of a ‘no treatment’ option (NICE, 2019). When exploring the various preoperative, intraoperative and postoperative complications associated with orthognathic surgery (Kim, 2017; Sousa and Turrini, 2012), clinicians may be asked, ‘What will happen if I don’t have surgery?’ Disappointingly, despite being presented in the 1980s, questions regarding the long-term effects of severe malocclusion on oral health, including whether surgical correction leads to an improvement, remain largely unanswered (Shaw et al., 1986). To the authors’ knowledge, there are no review articles that examine the subject comprehensively without eliminating large amounts of data. The critical informed consent process may therefore rely more upon anecdote and the personal experience of the surgical team than the complete and methodical interpretation of the best of the available research evidence.

The aim of the present article was to improve the orthognathic shared decision-making process for the clinician and patient by identifying and applying existing evidence to establish the potential consequences of living with a severe malocclusion.

## Methods

The methodology of a narrative literature review was adopted and combined with a deductive approach to identify, analyse and present all the relevant and available research evidence reporting on the consequences of severe malocclusion.

**Figure 1.**
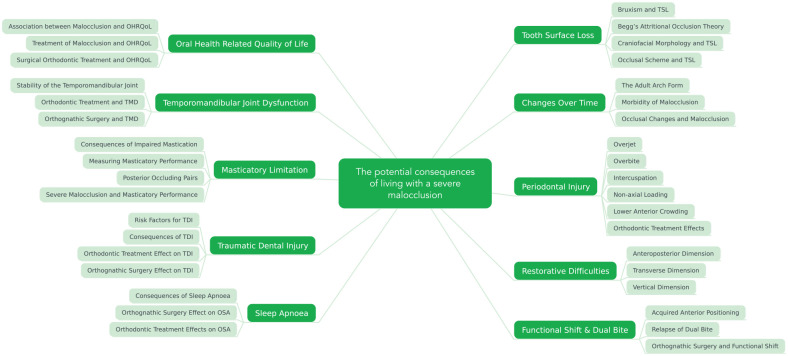
The potential consequences of living with a severe malocclusion.

A preliminary review of the literature identified a range of measures in which patients with severe malocclusions who would benefit from, but do not receive, orthognathic surgery may be potentially disadvantaged compared to those that undergo intervention and those with naturally occurring ideal occlusions. However, no review articles in which the subject is comprehensively investigated were found and so the research proposal, informed by the measures identified, was presented to consultant oral and maxillofacial surgeons, consultant orthodontists, academic clinical fellows and orthodontic specialty trainee registrars at University research meetings. Key words were developed via open discussion until 10 research themes emerged, as shown in Figure 1. Search terms: shown in Table 1, were formed in collaboration with a Senior University Health Sciences Research Librarian in order to ensure that all relevant studies and themes were included. A systematic electronic literature search of four online databases, as shown in Table 2, was conducted during March 2020. To ensure reliability, searches were performed independently by two researchers (RL and DB) and results were combined, incorporating all study designs in order to identify the highest level of evidence within each theme. Where evidence was limited, both researchers hand-searched reference lists obtained in the primary search, in order to generate additional articles of interest. All articles of interest were screened for relevance and quality by both of the researchers, and following discussion, the studies that offered the highest level of evidence within each theme were retained for inclusion in a comprehensive narrative literature review. A deductive approach was used to draw conclusions from the evidence, and checklists from the Critical Appraisal Skills Programme (CASP, 2019) and Centre for Evidence-Based Medicine (CEBM, 2015) were modified and combined with the Oxford Levels of Evidence table (OCEBM, 2011) to grade the supporting articles. A table summarising the appraisal, including the level of evidence, overall risk of bias, presence of statistical issues, the quality of reporting and intervention and the generalisability of the literature supporting each of the conclusions is available to view as online supplemental material.

**Table 1.** Systematic electronic literature search terms and limits.

	Base search terms			Theme	Limits
**OR**	Severe malocclusion	**AND**	**OR**	Oral health related quality of life	Human
	Orthognathic surgery			Self-worth	Medical
	Untreated			Self-value	Dental
	Refused			Self-esteem	English language
			**OR**	Temporomandibular joint dysfunction	
				Craniomandibular disorders	
				Temporomandibular disorder	
				Temporomandibular joint disorders	
				Temporomandibular joint dysfunction syndrome	
				Masticatory muscles	
				TMD	
				Jaw pain	
			**OR**	Masticatory limitation	
				Masticatory dysfunction	
				Nutrition	
				Diet	
			**OR**	Sleep apnoea	
				Obstructive sleep apnoea	
				Hypopnea	
				Sleep disturbance	
			**OR**	Traumatic dental injury	
				TDI	
				Dental trauma	
				Tooth injury	
			**OR**	Tooth wear	
				Tooth surface loss	
				TSL	
				Bruxism	
				Attrition	
			**OR**	Changes over time	
				Malocclusion changes	
				Worse with age	
				Worsening	
			**OR**	Periodontal injury	
				Soft tissue injury	
				Gingival surface injury	
				Gingival stripping	
				Recession	
				Traumatic occlusion	
				Idiopathic trauma	
				Akerly	
			**OR**	Restorative management	
				Restorative rehabilitation	
				Complications	
				Difficulties	
				Denture fabrication	
			**OR**	Functional shift	
				Dual bite	
				Acquired anterior positioning	
				Relapse of dual bite	
				Postured bite	

TDI, traumatic dental injury; TMD, temporomandibular joint dysfunction; TSL, tooth surface loss.

**Table 2.** Results of the systematic electronic literature search.

Theme	Scopus	Web of Science	MEDLINE	EMBASE	Articles of interest	Bibliography search	Included in review
	Whole collection	Whole collection	1946–2020 week 4	1980–2020 week 4			
OHRQoL	46	45	67	67	29	21	50
TMD	149	26	10	10	25	57	82
Masticatory Limitation	42	15	3	3	21	63	84
Sleep Apnoea	60	6	0	0	16	72	88
TDI	81	35	12	12	15	33	48
TSL	38	16	0	0	11	122	133
Changes Over Time	6	6	2	2	9	80	89
Periodontal Injury	81	35	12	12	9	93	102
Restorative Difficulties	119	2	0	0	8	33	41
Functional Shift and Dual Bite	1	1	1	1	3	79	82

OHRQoL, oral health related quality of life; TDI, traumatic dental injury; TMD, temporomandibular joint dysfunction; TSL, tooth surface loss.

## Results

A wide range of study designs were identified with well-developed research areas like temporomandibular joint dysfunction returning multiple high-quality systematic reviews while less established areas such as the Restorative Difficulties theme offered only individual case reports and expert opinion. Of the 1024 articles screened, 799 were examined closely in the comprehensive narrative literature review. The 27 conclusions generated are summarised and presented below.

### Oral Health Related Quality Of Life

Oral health related quality of life (OHRQoL) has been defined as ‘*the impact of oral disorders on aspects of everyday life that are important to patients and persons, with those impacts being of sufficient magnitude, whether in terms of severity, frequency or duration, to affect an individual’s perception of their life overall*’ (Locker and Allen, 2007).

An improvement in OHRQoL after surgical-orthodontic management of severe malocclusion has been demonstrated by several longitudinal studies (Alanko et al., 2017; Hatch et al., 1998; Lee et al., 2008; Motegi et al., 2003; Nicodemo et al., 2008). Recently, two systematic reviews concluded that the evidence supports an improvement in the aesthetic, functional, social and psychological aspects of OHRQoL for patients undergoing orthognathic surgery (De Araujo et al., 2020; Zamboni et al., 2019).

Adults with severe malocclusion or dentofacial malformation have poorer OHRQoL than those with normal occlusions (Frejman et al., 2013; Hassan and Amin, 2010; Lee et al., 2007).

### Temporomandibular Joint Dysfunction

Temporomandibular joint dysfunction (TMD) is a collective term for numerous dental, surgical, medical and psychological clinical problems involving the masticatory muscles, temporomandibular joints and associated structures (De Leeuw and Klasser, 2008).

Severe malocclusion and dentofacial malformation often occur in conjunction with TMD (Abrahamsson et al., 2013), which may cause adults to seek treatment (Alanko et al., 2014; Forssell et al., 1998). Indeed, TMD remains one of the principal complaints among those referred for orthognathic surgery (Magnusson et al., 1986; Rivera et al., 2000), reportedly occurring in 43%–73% of orthognathic cases (Onizawa et al., 1995; Panula et al., 2000; Westermark et al., 2001).

Patients with severe malocclusions may be more likely to develop TMD and an accurate pre-treatment TMD diagnosis is crucial (Abrahamsson et al., 2013; Celić et al., 2002; Egermark et al., 2003; Miller et al., 2004).
Patients with severe malocclusion and impaired masticatory performance diagnosed with TMD may benefit from orthognathic treatment (Abrahamsson et al., 2009, 2013); however, neither the presence of preoperative symptoms of TMD or the type of jaw deformity can identify which patients will improve, remain the same or worsen after surgery (Al-Moraissi et al., 2017), and no guarantees should be made (Al-Riyami et al., 2009).

### Masticatory Limitation

The mechanical reduction of food into smaller pieces facilitates the enzymatic processing of the digestive system by increasing surface area. Normal mastication requires coordinated activity of the teeth, salivary glands, tongue and muscles of mastication. Dysfunction in any area can impair masticatory function (N’Gom and Woda, 2002), resulting in suboptimal digestion (Kay and Sheine, 1979). Poor masticatory performance has been directly linked to the development of gastritis, ulcers and gastric carcinoma (Paul and Poitras, 1992), and a reduced dietary range has been shown to be associated with malnutrition (Krall et al., 1998).

● An individual whose malocclusion is severe enough to require orthodontic treatment might swallow larger food particles than one without need for orthodontic treatment (English et al., 2002; Ngom et al., 2007).
● A severe malocclusion may give rise to mechanical disadvantage, which, if an individual is unable to successfully adapt their masticatory technique, may negatively impact masticatory performance and OHRQoL (Bourdiol et al., 2017).
An untreated, severe malocclusion may result in masticatory limitation in later life, especially if occlusal contacts are subsequently reduced due to tooth loss (Abrahamsson et al., 2014; Hennequin et al., 2015; Magalhães et al., 2010).

### Sleep Apnoea

Obstructive sleep apnoea (OSA) is part of a spectrum of sleep disorders involving increased upper airway resistance during sleep (Panossian and Daley, 2013), and is characterised by recurrent partial or complete closure of the upper airway, despite ongoing efforts to breathe (Yim et al., 2006).

Signs and symptoms of OSA include frequent silences during sleep due to breaks in breathing, choking, gasping, snoring, sudden arousals, waking in a sweat, daytime fatigue, and an increased heart rate or elevated blood pressure (Malhotra and White, 2002).

● Dentofacial malformation and severe malocclusion can affect the development and maintenance of the airway (Cistulli, 1996).
Patients with severe malocclusions may be more prone to developing OSA (Reiche-Fischel and Wolford, 1996; Yu et al., 1994).
● Orthognathic surgery plays an important role in the management of OSA, though the effects on the posterior airway space are variable (Goodday et al., 2016).
Patients with severe malocclusions and undiagnosed, but compromised, airways may develop OSA in later life, and those with pre-existing OSA may find that the condition worsens beyond 65 years of age (Foley et al., 1995).

### Traumatic Dental Injury

A traumatic dental injury (TDI) is an impact injury to the teeth and or the hard and soft tissues within and around the vicinity of the mouth and oral cavity. It is usually sudden, circumstantial, unexpected or accidental, and may require emergency attention. It is not a disease but a consequence of potentially unavoidable risk factors in everyday life (Lam, 2016). A recent systematic review and meta-analysis of observational studies found that a large overjet may double or even triple the risk of TDI to anterior primary and permanent teeth, and at a global level an increased overjet is thought to be at least partly responsible for between 100 and 300 million TDIs (Petti, 2015).

In the permanent and primary dentition, an overjet >5 mm and 3 mm, respectively, represents a threshold for increased risk of TDI (Arraj et al., 2019).

### Tooth Surface Loss

Tooth surface loss (TSL) describes the irreversible destruction of dental hard tissue that occurs as a result of combined non-carious physiological or pathological processes (Bassiouny, 2012). Physiological TSL may affect the occlusal and incisal surfaces as a consequence of mastication, or the interproximal tooth surfaces as a result of friction generated between adjacent teeth (Davies et al., 2002). TSL is regarded as pathological if the rate of wear is greater than that expected for the patient’s age, if the patient experiences symptoms or if the prognosis of a tooth is compromised by the extent of the wear (Kelleher and Bishop, 1999). The process of pathological TSL is complex and multifactorial, but is usually described as a combination of attrition, abrasion, abfraction or erosion (Shellis and Addy, 2014).

The extent that a malocclusion contributes to the development and magnitude of TSL remains unclear (Bernhardt et al., 2004; Dahl et al., 1989; Warren et al., 2002), as both normal occlusions and severe malocclusions can demonstrate varying patterns and intensities of TSL (Janson et al., 2010).

● Most deviations in occlusal traits have not been shown to be significantly associated with TSL (Mwangi et al., 2009; Rugh et al., 1984; Seligman et al., 1988).
In some instances, anterior and unilateral posterior crossbites, and anterior crowding were protective of severe TSL (Berge et al., 1996; Bernhardt et al., 2004), while in others, they appear causative (Roberts-Harry and Sandy, 2003).
● Edge-to-edge and cusp-to-cusp relationships of teeth (Bernhardt et al., 2004), overbites >4 mm (Ritchard et al., 1992; Silness et al., 1993), and the Angle Class II malocclusion (Carlsson et al., 2003; Cunha-Cruz et al., 2010) are associated with higher levels of TSL.

### Changes Over Time

Although the attainment of biologic maturity in adulthood is often perceived as a period of no change, or possibly one of slow deterioration, many researchers have suggested that growth and development persists into and continues throughout adulthood (Baer, 1956; Behrents, 1986; Harris et al., 1992; Hrdlička, 1936; Lazenby, 1990a, 1990b).

It is reasonable to assume that continued skeletal growth may cause a malocclusion to change over time; however, as they occur slowly and continue for many decades, substantial longitudinal studies are required in order to demonstrate measurable changes to a malocclusion.

● The occlusion should be regarded as a dynamic rather than a stable interrelationship between facial structures (Bishara et al., 1989, 1994; Sillman, 1964; Sinclair and Little, 1983).
Patients with severe malocclusions may have fewer teeth at age 65, when compared with those with a normal occlusion in childhood (Stenvik et al., 2011).
Dissatisfaction associated with dental appearance when living with a severe malocclusion may lead to dental neglect (Disha et al., 2017; Hörup et al., 1987; Masood et al., 2013).

### Periodontal Injury

Periodontitis is not a single homogeneous condition but instead, a family of closely related diseases each of which may vary in aetiology, natural history and response to therapy (Page and Schroeder, 1982). The resultant clinical condition is influenced by and is the sum of, genetic, environmental or acquired systemic disease modifiers (Page and Kornman, 1997).

Historically, a simple justification for orthodontic treatment was that irregular teeth increase one’s susceptibility to periodontitis as they are more difficult to clean. Indeed, some authors have suggested a substantial relationship between malocclusion and periodontitis (Alexander, 1970; Buckley, 1981; Hellgren, 1956; Poulton and Aaronson, 1961; Sandalli, 1973), yet others have found no significant association (Ainamo, 1972; Beagrie, 1962; Geiger, 1962; Katz, 1978).

● For patients with severe deep bite malocclusion, gingival surface injury can result in substantial and irreversible damage to the periodontium over time (Brook and Shaw, 1989).
Sustained occlusal trauma may result in reduction of alveolar bone density and widening of the periodontal ligament space (Comar et al., 1969).
● A severely increased overjet, in combination with mouth breathing, or the absence of lip coverage, may increase the prevalence of gingivitis around the incisor teeth (Jacobson and Linder-Aronson, 1972; Wagaiyu and Ashley, 1991).
● The presence of non-working side contact is associated with deeper probing depth and more clinical attachment loss (Bernhardt et al., 2006); however, orthodontic correction of these contacts may reduce the progression of periodontitis or improve the prognosis of periodontal therapy in those who develop periodontal disease in later life.
● Severe mandibular incisor crowding and irregularity are associated with periodontal disease progression in later life (Alsulaiman et al., 2018).
● If a satisfactory occlusal stop is not established, overbite can continue to increase even after successful orthodontic treatment (Binda et al., 1994; Canut and Arias, 1999;).

### Restorative Difficulties

Although orthodontic treatment is frequently discussed in the prosthodontic literature, it is generally with reference to implant planning, or limited to the enhancement of anterior aesthetics (Goodacre et al., 1997; Miller, 1989). While prosthodontic treatment can camouflage some minor occlusal discrepancies, complex restorative problems that arise as a result of severe malocclusion will generally benefit from pre-prosthetic orthodontic therapy (Bidra and Uribe, 2012). Still, despite offering the potential for more stable, durable and aesthetic treatments (Goodacre et al., 1997; Miller, 1995), few studies describe the use of orthodontic therapy before restorative rehabilitation (Cohen, 1995).

A severe malocclusion in any dimension will complicate restorative and prosthodontic management for the dentist and dental technician. Without multidisciplinary surgical management, the functional stability and aesthetic outcome of a prosthodontic rehabilitation for those with severe skeletal discrepancies is often a compromise (Pektas and Kircelli, 2014).
The strategic pre-prosthetic orthodontic treatment of a patient with a severe malocclusion before full-mouth restorative rehabilitation offers numerous advantages (Goodacre et al., 1997; Miller, 1995).

### Functional Shift and Dual Bite

Although the terms are often used interchangeably in the literature, functional shift of the mandible describes an occlusally determined positional change in an anteroposterior or lateral direction. It is typically associated with a transverse skeletal discrepancy or occlusal interference, which results in an involuntary retruded contact position to intercuspal position (RCP-ICP) discrepancy of >2 mm (Ishizaki et al., 2010; Nerder et al., 1999; Severt and Proffit, 1997). Dual bite, or ‘Sunday bite’, describes a voluntary or aesthetically determined anteroposterior positional change from RCP to a second, more anterior or lateral occlusal position (Posselt, 1968).

When in their respective physiological rest positions, patients with a class II malocclusion posture into a more protrusive rest-position than patients with a class I occlusion (Curtis et al., 1988; Emrich et al., 1965; Lowe et al., 1983). This elective and adaptive posturing improves aesthetics (Dierkes, 1987), lip seal, muscle function, speech and respiration, and accommodates the underlying skeletal relationship (Gibbs et al., 1984; Owen III, 1998; Ricketts, 1956). The effect of failing to correct functional shift with orthodontics is scarcely discussed in the literature. However, there is limited evidence to suggest that chronic anterior posturing of the mandible may cause overeruption of the posterior teeth, a clockwise autorotation of the mandible around molar fulcrums, occlusal re-interdigitation with the condyles seated in the acquired anterior position and development of an anterior open bite (Tamimi and Hatcher, 2016).

● Patients with severe class II and class III malocclusion have shown to be more likely to adopt anterior and posterior postural changes in order to improve masticatory function and facial aesthetics (Sperry, 1989).
● Chronic anterior posturing of the mandible can lead to the ‘relapse’ of a dual bite and development of an acquired anterior open bite (Tamimi and Hatcher, 2016).

## Discussion

When following a well-designed protocol and addressing a clinically relevant and focused question, systematic review methodology may strengthen or clarify previously published conclusions and increase statistical power through collective analysis (Glenny et al., 2003). However, when the primary evidence exhibits diverse interventions and study populations or varying methodologic design and quality, pooling is inappropriate as it risks formation of unreliable estimates and a false sense of precision (Lau et al., 1998). Recently, when researchers examined the impact of malocclusion and orthodontic treatment on oral health, they reported that the heterogeneity of studies included in their systematic review constrained the development of conclusions and resulted in the rejection of large amounts of clinically relevant and useful information (Macey et al., 2020). It remains impractical, however, for clinicians to locate and examine every primary study. In order to address the aim of this project and identify, analyse and present all of the relevant evidence in a comprehensive summary that incorporates uncertainty without excluding it from review while drawing meaningful conclusions from an extensive but non-homogenous evidence base, the narrative literature review combined with a deductive approach was deemed the optimal study design.

After a review of 799 articles, the authors identified a range of complications that may impact upon those living with a severe malocclusion, yet the literature that supports the majority of the 27 conclusions reached was found to be of low to moderate quality and at high risk of bias.

In agreement with preceding studies (Arraj et al., 2019; Javidi et al., 2017; Macey et al., 2020), the highest-quality evidence was found within the OHRQoL (De Araujo et al., 2020; Zamboni et al., 2019) and TDI (Arraj et al., 2019) themes, indicating that these conclusions may be discussed with patients with a high degree of confidence. High-quality but conflicting evidence was available within the TMD theme (Al-Moraissi et al., 2017; Al-Riyami et al., 2009), where despite the suggestion of a potential trend, the contradictory nature of the evidence led to uncertainty, reducing the applicability of the conclusions. The majority of the evidence in support of the Masticatory Limitation, OSA, TSL and Changes Over Time themes was of low to moderate quality, with deficiencies in reporting (Ritchard et al., 1992; Yu et al., 1994) and statistical issues such as absence of a sample size calculation (Berge et al., 1996; Ngom et al., 2007; Reiche-Fischel and Wolford, 1996; Stenvik et al., 2011). Consequently, while these conclusions add to the current knowledge base, they are potentially less significant, and patients should be made aware that further studies are required to validate them. When exploring the Restorative Difficulties, Periodontal Injury and Functional Shift and Dual Bite themes, evidence was scarce and conclusions were limited to inference derived from individual case reports (Pektas and Kircelli, 2014), case series (Miller, 1995), expert opinion (Goodacre et al., 1997; Tamimi and Hatcher, 2016) and an animal experimental study (Comar et al., 1969). The decision to incorporate these conclusions into the shared decision-making process is therefore more contentious as modern, high-quality systematic review methodology would disregard the supporting evidence and clinicians should make this clear if discussing them with patients.

When making the decision to include conclusions based on such limited evidence in this review, the authors drew parallels to the significant variation of opinion that exists within the respective fields of clinical orthodontics and orthodontic research. A malocclusion that one clinician may consider an orthognathic approach unavoidable, may be successfully managed with orthodontics by another, for example. Similarly, evidence-based medicine encourages a ‘bottom-up’ approach that, in the absence of higher quality studies, integrates the best available evidence with individual clinical expertise and patient choice, proposing that, ‘*if no randomised trial has been carried out for our patient’s predicament, we must follow the trail to the next best external evidence and work from there*’ (Sackett et al., 1996). External evidence may therefore inform but not replace individual clinical expertise and experience, and with this in mind, it is the author’s opinion that clinicians should have the opportunity first to decide whether the presented evidence is relevant and applicable to their individual patient and secondly whether the quality of evidence is sufficient for it to influence a clinical decision.

## Conclusion

Although the conclusions presented within this article cannot be considered as certainties, when combined with an expert understanding of the underlying malocclusion and an awareness of the patient’s individual preferences and opinions, they may provide a useful foundation for the shared decision-making process, helping patients to understand the potential implications of declining surgical intervention while ensuring that clinicians fulfil their obligations regarding informed consent. In addition, the findings of this review may guide the development of further high-quality longitudinal research required to validate the potential consequences of living with a severe malocclusion.

## Supplemental Material

sj-pdf-1-joo-10.1177_14653125211042891 – Supplemental material for The consequences of living with a severe malocclusion: A review of the literature Click here for additional data file. Supplemental material, sj-pdf-1-joo-10.1177_14653125211042891 for The consequences of living with a severe malocclusion: A review of the literature by Richard Leck, Ninu Paul, Sarah Rolland and David Birnie in Journal of Orthodontics
