# Supplementary material for: The consequences of living with a severe malocclusion: A review of the literature
Source: J Orthod. 2021 Sep 6;49(2):228–39. doi: 10.1177/14653125211042891 (PMC9160782; doi:10.1177/14653125211042891)
Supplement: sj-pdf-1-joo-10.1177_14653125211042891 – Supplemental material for The consequences of living with a severe malocclusion: A review of the literature [file sj-pdf-1-joo-10.1177_14653125211042891.pdf]

Appraisal of the evidence supporting the conclusions derived from the comprehensive narrative literature review

				Study Details			Quality Assessment			Additional Considerations				
	Conclusion	Author & Year	Journal	Country	Sample Size	Design	Evidence Level	Appropriate Design	Risk of Bias	Outcome Measures	Statistical Issues	Quality of Reporting	Quality of Intervention	Generalisable
OHRQoL	Adults with severe malocclusion or dentofacial malformation have been shown to have poorer OHRQoL than those with normal occlusions	*Frejman et al. 2013*	*J Oral Maxillofac Surg*	Brazil	68	Cross sectional observational	**3**	Yes	Moderate	Rosenberg Self-Esteem Scale, General Hospital Depression Scale	No	Good	High	Yes
		Hassan and Amin 2010	*Am J Orthod Dentofacial Orthop*	Saudi Arabia	366	Cross sectional observational	**3**	Yes	Moderate	DHC of the IOTN, OHIP-14	Yes	Good	Moderate	No
		Lee et al. 2007	*Int J Oral Maxillofac Surg*	Hong Kong	152	Case-control	**3**	Yes	High	SF-36, OHIP-14, OQLQ-22	Yes	Good	Moderate	Yes
Temporomandibular Joint Dysfunction	Patients with severe malocclusions may be more likely to develop TMD therefore an accurate pre-treatment TMD diagnosis is of paramount importance	*Abrahamsson et al. 2013*	*Int J Adult Orthodon Orthognath Surg*	Sweden	121	Case control	**3**	Yes	Moderate	RDC/TMD	No	Good	Moderate	Yes
		Celić et al. 2002	*Int J Prosthodont*	Croatia	230 Males	Observational	**3**	No	High	History, clinical examination	Yes	Moderate	Low	No
		Egermark et al. 2003	*Angle Orthod*	Sweden	320	Longitudinal	**3**	Yes	Moderate	Clinical examination, Helkimo index	Yes	Good	Moderate	Yes
		Miller et al. 2004	*J Public Health Dent*	USA	311 Females	Case control	**3**	No	High	Questionnaire, sagittal photographs	Yes	Moderate	Moderate	No
	Patients with severe malocclusion and impaired masticatory performance diagnosed with TMD may benefit from orthognathic treatment, however, neither the presence of presurgical TMD symptoms or the type of jaw deformity can identify which patients' TMDs would improve, remain the same, or worsen after surgery, and no guarantees should be made	*Abrahamsson et al. 2009*	*Angle Orthod*	Sweden	121	Case control	**3**	Yes	Low	RDC/TMD	No	Good	Moderate	Yes
		Abrahamsson et al. 2013	*Int J Adult Orthodon Orthognath Surg*	Sweden	121	Case control	**3**	Yes	Moderate	RDC/TMD	No	Good	Moderate	Yes
		Al-Moraissi et al. 2017	*J Oral Maxillofac Surg*	Yemen, USA	29 Studies	Systematic review and meta analysis	**1a**	Yes	Low	Questionnaire, clinical examination, RDC/TMD, Helkimo index, amnestatic dysfunction index	No	Good	High	Yes
		Al-Riyami et al. 2009	*J Oral Maxillofac Surg*	UK	5 studies	Systematic review and meta analysis	**1a**	Yes	Low	Helkimo index, self reported, clinical examination	No	Good	High	Yes
Masticatory Limitation	A subject whose malocclusion is severe enough to require orthodontic treatment might swallow larger food particles than one without need for orthodontic treatment	*English et al. 2002*	*Angle Orthod*	USA	147	Case control	**3**	Yes	High	Cumulative weight percentages of silicone sample, chewing performance with natural foods, questionnaire	Yes	Good	High	Yes
		Ngom et al. 2007	*Am J Orthod Dentofacial Orthop*	Senegal	102	Case control	**3**	Yes	Moderate	ICON, Frequency of mastication, median particle size, broadness of particle distribution	Yes	Moderate	Moderate	No
	A severe malocclusion may give rise to mechanical disadvantage, which, if an individual is unable to successfully adapt their masticatory technique, may in turn negatively impact masticatory performance and QoL	*Bourdiol et al. 2017*	*Arch Oral Biol*	France	42	Case control	**3**	Yes	Moderate	Masticatory Normative Indicator, functional contacts, bolus granulometric analysis	No	Good	High	Yes
	An untreated, severe malocclusion may result in masticatory difficulties in later life, especially if occlusal contacts are subsequently reduced due to tooth loss	*Abrahamsson et al. 2014*	*Eur J Orthod*	Sweden	98	Longitudinal prospective controlled trial	**2**	Yes	Moderate	Masticatory performance test, self-estimated masticatory ability	Yes	Good	Moderate	Yes
		Hennequin et al. 2015	*Physiol Behav*	France	26	longitudinal prospective controlled trial	**2**	Yes	Moderate	Vineland Adaptive Behaviour Scale-survey, number of posterior functional units, kinematic parameters of chewing	Yes	Moderate	Moderate	No
		Magalhães et al. 2010	*Angle Orthod*	Brazil	12 studies	Systematic review	**1b**	Yes	Low	Masticatory performance, median particle size	N/A	Good	High	Yes
Sleep Apnoea	Dentofacial malformation and severe malocclusion can affect the development and maintenance of the airway	*Cistulli 1996*	*Respirology*	Australia	N/A	Review article	**5**	No	High	N/A	N/A	Moderate	N/A	N/A
	Patients with severe malocclusions may be more prone to developing OSA	*Reiche-Fischel and Wolford 1996*	*J Oral Maxillofac Surg*	USA	72	Case control	**3**	Yes	High	Degree of mandibular advancement, posterior airway space, % PAS change	Yes	Low	Low	No
		Yu et al. 1994	*J Oral Maxillofac Surg*	USA	26	Retrospective observational	**3**	No	High	Cephalometric parameters	Yes	Low	Moderate	Yes
	Orthognathic surgery plays an important role in the management of OSA, though the effects on PAS are variable	*Goodday et al. 2016*	*J Oral Maxillofac Surg*	Canada	265	Retrospective observational	**3**	No	Moderate	PSG data, self-administered questionnaire, CPAP usage	Yes	Moderate	Moderate	Yes
	Patients with severe malocclusions and undiagnosed, but compromised airways may develop OSA in later life and those with pre-existing OSA may find that the condition worsens beyond 65 years of age	*Foley et al. 1995*	*Sleep*	USA	10,294	Epidemiologic Study	**3**	Yes	Moderate	Interview	Yes	Moderate	Moderate	Yes
TDI	In the permanent and primary dentition, an overjet greater than 5 mm and 3 mm respectively represents a threshold for increased risk of TDI	*Arraj et al. 2019*	*Dent Traumatol*	Australia	41 studies	Systematic review and meta-analysis	**1a**	Yes	Low	Overjet	No	Good	Good	Yes
Tooth Surface Loss	Most deviations in occlusal traits have not been shown to be significantly associated with TSL	*Mwangi et al. 2009*	*Am J Orthod Dentofacial Orthop*	UK	307	Cross sectional	**3**	Yes	Moderate	Occlusal characteristics, Hooper Index	Yes	Moderate	Low	No
		Rugh et al. 1984	*J Prosthet Dent*	USA	10	Prospective controlled	**3**	No	High	EMG activity, questionnaire / reports	Yes	Moderate	Moderate	No
		Seligman et al. 1988	*J Dent Res*	USA	222	Cross sectional	**3**	Yes	High	History, clinical examination, study casts	Yes	Moderate	Moderate	Yes
	In some instances, anterior and unilateral posterior crossbites, and anterior crowding were protective of severe TSL, whilst in others, they appear causative	*Berge et al. 1996*	*J Oral Rehabil*	Norway	50	Cross sectional	**3**	No	High	NONAT, TWI	Yes	Low	Low	No
		Bernhardt et al. 2004	*J Periodontol*	Germany	2529	Cross sectional epidemiological	**3**	Yes	Moderate	Occlusal relationships, sociodemographic parameters, attrition index	Yes	Moderate	Moderate	No
		Roberts-Harry and Sandy 2003	*Br Dent J*	UK	N/A	Expert opinion	**5**	N/A	High	N/A	N/A	N/A	N/A	N/A
	Edge-to-edge and cusp-to-cusp relationships of teeth overbites greater than 4 mm and the Angle Class II malocclusions are associated with higher levels of TSL	*Bernhardt et al. 2004*	*J Periodontol*	Germany	2529	Cross sectional epidemiological	**3**	Yes	Moderate	Occlusal relationships, sociodemographic parameters, attrition index	Yes	Moderate	Moderate	No
		Ritchard et al. 1992	*Aust Orthod J*	Australia	298	Cross sectional	**3**	Yes	High	Occlusal relationships, attrition index	Yes	Low	Low	No
		Silness et al. 1993	*Acta Odontol Scand*	Norway	51	Longitudinal prospective controlled trial	**3**	Yes	Moderate	Overjet, overbite, occlusal wear index	Yes	Moderate	Moderate	No
		Carlsson et al. 2003	*J Orofac Pain*	Sweden	320	Longitudinal prospective controlled trial	**3**	Yes	Moderate	Occlusal relationships, subjective reports of bruxism	Yes	High	High	No
		Cunha-Cruz et al. 2010	*Community Dent Oral Epidemiol*	USA	1530	Cross sectional	**3**	Yes	High	Occlusal relationships, dental history, subjective reports of bruxism	Yes	Moderate	Moderate	Yes
Changes Over Time	The occlusion should be regarded as a dynamic rather than a stable interrelationship between facial structures	*Bishara et al. 1989*	*Am J Orthod Dentofacial Orthop*	USA	34	Longitudinal	**3**	Yes	Moderate	Cephalometric parameters, occlusal relationships	Yes	Moderate	High	Yes
		Bishara et al. 1994	*Am J Orthod Dentofacial Orthop*	USA	29	Longitudinal	**3**	Yes	Moderate	Cephalometric parameters, occlusal relationships	Yes	Moderate	High	Yes
		Sillman 1964	*Am J Orthod*	USA	65	Longitudinal	**3**	Yes	Moderate	Anatomical measurements, occlusal relationships	Yes	Moderate	Moderate	Yes
		Sinclair and Little 1983	*Am J Orthod*	USA	65	Longitudinal	**3**	Yes	Moderate	Anatomical measurements, occlusal relationships	Yes	Moderate	Moderate	Yes
	Patients with severe malocclusions experience more problems tooth-related problems in later life compared with those with a normal occlusion in childhood	*Stenvik et al. 2011*	*Am J Orthod Dentofacial Orthop*	Norway	69	Longitudinal	**3**	Yes	Moderate	Occlusal relationships	Yes	High	Low	Yes
	Dissatisfaction associated with dental appearance when living with a severe malocclusion may lead to dental neglect	*Disha et al. 2017*	*J Educ Health Promot*	India	800	Cross-sectional survey	**3**	Yes	High	Occlusal relationships, DMFT	Yes	Moderate	Low	No
		Hörup et al. 1987	*Community Dent Oral Epidemiol*	Norway	422	Cross-sectional survey	**3**	Yes	Moderate	Satisfaction with dentition, occlusal relationships, SDFT, plaque score, dental attendance history	Yes	Moderate	Moderate	Yes
		Masood et al. 2013	*Health Qual Life Out*	Malaysia	323	Cross-sectional survey	**3**	Yes	Moderate	OHIP-14, IOTN-DHC	Yes	Moderate	Moderate	Yes
Periodontal Injury	For patients with severe deep bite malocclusion, gingival surface injury can result in substantial and irreversible damage to the periodontium over time	*Brook and Shaw 1989*	*Eur J Orthod*	UK	222	Cross sectional	**3**	Yes	Low	IOTN DHC / AC, SCAN index	No	Moderate	Moderate	Yes
	Sustained occlusal trauma, may result in reduction of alveolar bone density and widening of the periodontal ligament space	*Comar et al. 1969*	*J Periodontol*	USA	4	Animal experimental study	**5**	No	High	Pocket depth, mobility, clinical presentation	N/A	Moderate	High	No
	A severely increased overjet, in combination with mouth breathing, or the absence of lip coverage, may increase the prevalence of gingivitis around the incisor teeth	*Jacobson and Linder-Aronson 1972*	*Eur J Oral Sci*	Sweden	95	Cross sectional	**3**	Yes	High	Interarch space differential, gingival index	Yes	Moderate	Moderate	No
		Wagaiyu and Ashley 1991	*J Clin Periodontol*	Kenya	201	Cross sectional	**3**	Yes	Moderate	Bleeding on probing, plaque score incisor crowding, mouth breathing, lip seal, coverage	Yes	Moderate	Moderate	No
	The presence of non-working side contact is associated with deeper probing depth and more clinical attachment loss, however, orthodontic correction of these contacts may reduce the progression of periodontitis or improve the prognosis of periodontal therapy in those who develop periodontal disease in later life	*Bernhardt et al. 2006*	*J Periodontol*	Germany	2980	Cross sectional epidemiological	**3**	Yes	Moderate	Medical examination, dental examination, interview, questionnaire	No	High	High	Yes
	Severe mandibular incisor crowding and irregularity are associated periodontal disease progression in later life	*Alsulaiman et al. 2018*	*Am J Orthod Dentofacial Orthop*	USA	804 Males	Retrospective longitudinal	**3**	No	Moderate	Pocket depth, alveolar bone loss, tooth presence	No	High	Low	No
	If a satisfactory occlusal stop is not established, overbite can continue to increase even after successful orthodontic treatment	*Binda et al. 1994*	*Eur J Orthod*	Netherlands	81	Longitudinal	**3**	Yes	Moderate	Cephalometric parameters	Yes	Moderate	Moderate	No
		Canut and Arias 1999	*Eur J Orthod*	Spain	30	Retrospective longitudinal	**3**	No	High	Occlusal characteristics	Yes	Moderate	Low	No
Restorative Difficulties	A severe malocclusion in any dimension will complicate restorative and prosthodontic management for the dentist and dental technician. Without multidisciplinary surgical management, the functional stability and aesthetic outcome of a prosthodontic rehabilitation for those with severe skeletal discrepancies is often a compromise	*Pektas and Kircelli 2014*	*J Prosthet Dent*	Turkey	1	Case report	**5**	Yes	High	N/A	N/A	Low	Low	No
	The strategic pre-prosthetic orthodontic treatment of a patient with a severe malocclusion prior to full-mouth restorative rehabilitation offers numerous advantages	*Goodacre et al. 1997*	*J Prosthet Dent*	USA	N/A	Expert opinion	**5**	N/A	High	N/A	N/A	N/A	N/A	N/A
		Miller 1995	*Dent Pract Dent Rec*	USA	3	Case series	**5**	Yes	High	N/A	N/A	Low	Low	No
Functional Shift & Dual Bite	Patients with severe class II and class III malocclusion have been shown to be more likely to adopt anterior and posterior postural changes in order to improve masticatory function and facial aesthetics	*Sperry 1989*	*Angle Orthod*	USA	85	Observational	**3**	Yes	High	Cephalometric parameters	Yes	Moderate	Moderate	Yes
	Chronic anterior posturing of the mandible can lead to overeruption of the primary or permanent posterior teeth, autorotation of the mandible around molar fulcrums, tooth re-interdigitation with the condyles seated in the acquired anterior position and an anterior open bite	*Tamimi and Hatcher 2016*	*Elsevier - textbook*	N/A	N/A	Expert opinion	**5**	N/A	High	N/A	N/A	N/A	N/A	N/A
